# Bildgebende Diagnostik des Dünndarms

**DOI:** 10.1007/s00117-025-01558-3

**Published:** 2026-01-19

**Authors:** Martina Scharitzer, Thomas Mang, Philipp Schreiner, Ulrike Attenberger

**Affiliations:** 1 Universitätsklinik für Radiologie und Nuklearmedizin, Medizinische Universität Wien, Währinger Gürtel 18–20, 1090 Wien, Österreich; 2 Universitätsklinik für Innere Medizin III, Medizinische Universität Wien, Wien, Österreich

**Keywords:** Dünndarmerkrankungen, Enterographie, Computertomographie, Magnetresonanztomographie, Intestinaler Ultraschall, Intestinal diseases, Enterography, Computed tomography, Magnetic resonance imaging, Intestinal ultrasound

## Abstract

Schnittbildverfahren spielen eine zentrale Rolle bei der Untersuchung des Jejunums und des Ileums, da diese Abschnitte im Gegensatz zu Magen, Duodenum und Kolon endoskopisch weniger leicht zugänglich sind. Die radiologische Diagnostik des Dünndarms stellt jedoch eine besondere Herausforderung dar, da viele Dünndarmerkrankungen ähnliche Bildmuster aufweisen und sich daher nicht immer einfach voneinander unterscheiden lassen. Technische Fortschritte in der Bildgebung ermöglichen eine noch präzisere Charakterisierung der Darmwand und schaffen damit die Grundlage für eine zunehmend personalisierte Medizin. Ein bildmusterbasierter diagnostischer Ansatz hilft dabei, die Zahl möglicher Diagnosen einzugrenzen und die radiologische Beurteilung zu präzisieren. Ziel dieser Übersichtsarbeit ist es, aktuelle diagnostische Verfahren, neueste Entwicklungen und charakteristische radiologische Zeichen von Dünndarmerkrankungen darzustellen.

## Abstract (en)

Cross-sectional imaging procedures play a pivotal role in the assessment of the jejunum and ileum as these segments of the small intestine are less easily accessible to endoscopic evaluation compared to the stomach, duodenum and colon. The radiological diagnostics of the small bowel are, however, a special challenge as many small bowel diseases show similar imaging patterns making a differentiation difficult. Technical advances in imaging now enable a more precise characterization of the bowel wall, thereby providing the basis for increasingly more personalized medicine. An imaging pattern-based diagnostic approach can be helpful in narrowing down the spectrum of potential differential diagnoses and increasing the accuracy of radiological interpretation. The aim of this review article is to present the current diagnostic techniques, recent technological developments and characteristic radiological imaging features of small bowel diseases.

## Lernziele

Nach Absolvieren dieser Fortbildungseinheit …

- haben Sie einen Überblick über moderne bildgebende Verfahren wie den intestinalen Ultraschall, die CT(Computertomographie)- und MR(Magnetresonanz)-Enterographie zur Diagnose und Beurteilung von Dünndarmerkrankungen.
- kennen Sie die Grundlagen zur technischen Durchführung sowie standardisierte Untersuchungsprotokolle verschiedener Schnittbildverfahren des Dünndarmes und können diese indikationsgerecht einsetzen.
- können Sie einen strukturierten Ansatz anwenden, der das morphologische Erscheinungsbild der Darmwandverdickung, deren Ausmaß sowie die Symmetrie und die Länge des betroffenen Darmabschnitts umfasst, um mögliche Differenzialdiagnosen einzugrenzen.
- haben Sie Kenntnis, wie Dünndarmwandveränderungen in der Schnittbilddiagnostik anhand pathologischer Muster und charakteristischer Bildgebungsmerkmale zuverlässig identifiziert werden können.

## Einleitung

Die radiologische Dünndarmbildgebung hat durch technologische Fortschritte erheblich an Bedeutung gewonnen und ist ein zentrales Verfahren sowohl in der Akutdiagnostik als auch zur Beurteilung chronischer Erkrankungen. Aktuelle Leitlinien empfehlen daher **Schnittbildverfahren**

Schnittbildverfahren

als Methode der Wahl für Diagnose, Staging und Verlaufsbeurteilung [1, 2]. Eine systematische Bildanalyse unterstützt die Darmwandcharakterisierung und die gezielte Eingrenzung möglicher Differenzialdiagnosen. Viele radiologische Muster und Zeichen sind jedoch unspezifisch und müssen im Kontext mit der klinischen Symptomatik und Anamnese der Patient:innen interpretiert werden.

## Kurzkasuistik

Eine 33-jährige Patientin mit floridem **Morbus Crohn**

Morbus Crohn

und **Ileitis terminalis**

Ileitis terminalis

mit fehlendem Ansprechen auf mehrere Biologika erhält eine Ileozökalresektion. Postoperativ erfolgt eine 3‑monatige prophylaktische antibiotische Therapie mit Metronidazol. Wenige Wochen nach Absetzen der Antibiose kommt sie mit moderaten Bauchschmerzen in die gastroenterologische Ambulanz. Es wird eine Sonographie des Abdomens einschließlich des Darms durchgeführt.

**Abb. 1:**
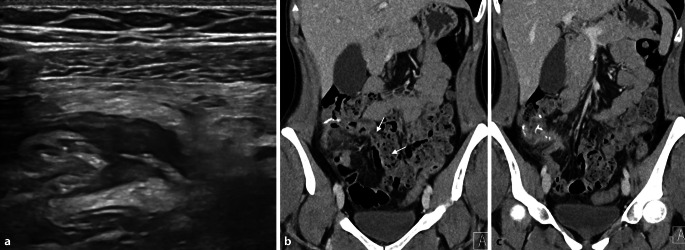
Die Sonographie des rechten Oberbauchs (**a**) mit einem hoch auflösenden Linearschallkopf zeigt das blinde Ende der Seit-zu-Seit-Enterokolostomie. **b,c** Venöse Computertomographie des Abdomens mit Nahtmaterial im Bereich der Anastomosenregion, **b** Verlauf des neoterminalen Ileums (*Pfeile*)

Im Anschluss erfolgt eine Computertomographie (CT) des Abdomens mit intravenösem Kontrastmittel (Abb. 1).

## Diagnostik

Bei der diagnostischen Abklärung von Dünndarmerkrankungen nimmt die Schnittbildgebung eine zentrale Rolle ein. Dabei kommen hoch auflösende Verfahren wie die Sonographie (**intestinaler Ultraschall**

Intestinaler Ultraschall,

IUS) und die **Magnetresonanztomographie**

Magnetresonanztomographie

(MRT) sowie für die Akutdiagnostik auch die **CT**

CT

zum Einsatz. Ein direkter Vergleich zwischen Ultraschall und MRT bei Patienten mit M. Crohn zeigte in der prospektiven METRIC-Studie eine hohe Sensitivität für beide Verfahren und bestätigte deren Einsatz als Erstuntersuchung. Allerdings wies die MRT bei Patienten mit Erstdiagnose eines M. Crohn sowohl für den Nachweis einer Dünndarmerkrankung als auch für deren segmentale Lokalisation eine signifikant höhere Sensitivität und Spezifität (96 und 99 %) als die Sonographie (77 und 98 %) auf [3]. Eine systematische Übersichtsarbeit von Bollegala et al. [4] ergab für den IUS eine Sensitivität zwischen 54 und 93 % bei der Diagnostik von M. Crohn des Dünndarms, wobei die Sensitivität bei milden Verlaufsformen geringer war. Der intestinale Ultraschall bietet dabei im Vergleich zur MRT den Vorteil der breiten Verfügbarkeit und unmittelbaren Durchführbarkeit. Ergänzend stehen als Zweitlinienmodalitäten endoskopische Verfahren wie die **Doppelballonenteroskopie**

Doppelballonenteroskopie

oder die **Kapselendoskopie**

Kapselendoskopie

und Hybridverfahren wie **PET(Positronenemissionstomographie)-CT**

PET-CT

und **PET-MRT**

PET-MRT

zur Verfügung.

### Abdomen-Leeraufnahme

Die Röntgenaufnahme des Abdomens hat mit nur 49 % eine moderate Sensitivität für die Diagnostik einer intestinalen Obstruktion [5]. Sie ist der Abdomen-CT signifikant unterlegen [6]. Daher wird die Röntgenaufnahme bei unklaren abdominalen Schmerzen und Verdacht auf intestinale Obstruktion nicht als primäre bildgebende Untersuchung empfohlen [7]. Indikationen für eine Abdomen-Leeraufnahme bestehen beim Dünndarm hingegen in der Suche nach intestinalen metalldichten Fremdkörpern, der Beurteilung der Passage eines oral verabreichten, positiven Kontrastmittels bei Verdacht auf eine inkomplette Obstruktion oder bei intestinaler Paralyse sowie in der Verlaufskontrolle einer bereits bekannten Pneumatosis intestinalis.

### Sonographie

Mittels moderner Ultraschalltechnologien lassen sich Darmstrukturen sonographisch in hoher Detailgenauigkeit darstellen. Limitierend sind eine unzureichende methodische Erfahrung und die fehlende standardisierte Abbildung aller Dünndarmsegmente. Darüber hinaus kann die Aussagekraft bei ausgeprägtem Meteorismus, Adipositas, Obstipation oder bei Peritonitis mit starker Schmerzsymptomatik erheblich eingeschränkt sein. Die routinemäßige Gabe von oralem Kontrastmittel hat sich aufgrund der längeren Untersuchungsdauer, reduzierten Patientenakzeptanz und des begrenzten Zusatznutzens nicht durchgesetzt.

**Abb. 2:**
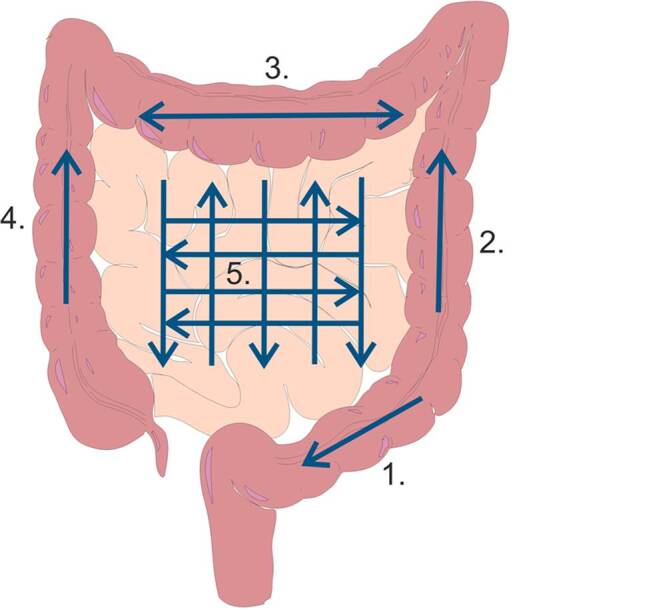
Schematische Darstellung eines standardisierten Ablaufs einer intestinalen Sonographie, der eine vollständige Untersuchung von Dünn- und Dickdarm gewährleistet

Um neben dem terminalen Ileum möglichst alle Anteile des Dünndarms zu erfassen, ist ein systematisches Untersuchungsprotokoll erforderlich. Dabei sollte der Bauchraum in parallel verlaufenden, sich überlappenden Bahnen in kranialer und kaudaler Richtung untersucht werden (Abb. 2). Mittels hoch frequenter Konvexschallköpfe (1–6 MHz) sowie Linearschallköpfe (12–15 MHz oder höher) können die Darmwanddicke, die Schichtstruktur der Darmwand und mittels **Dopplersonographie**

Dopplersonographie

auch die Vaskularisation beurteilt werden.

Zur semiquantitativen Klassifikation der Darmvaskularisation wird häufig der Limberg-Score herangezogen, besonders im Rahmen entzündlicher Veränderungen [8]. Dieser unterscheidet 5 Grade:

- Grad 0 (unauffällige Darmwand),
- Grad 1 (Wandverdickung ohne gesteigerte Vaskularisation),
- Grad 2 (kurzstreckige Gefäße),
- Grad 3 (langstreckige Gefäße),
- Grad 4 (multiple ins Mesenterium einstrahlende langstreckige Gefäße).

**Abb. 3:**
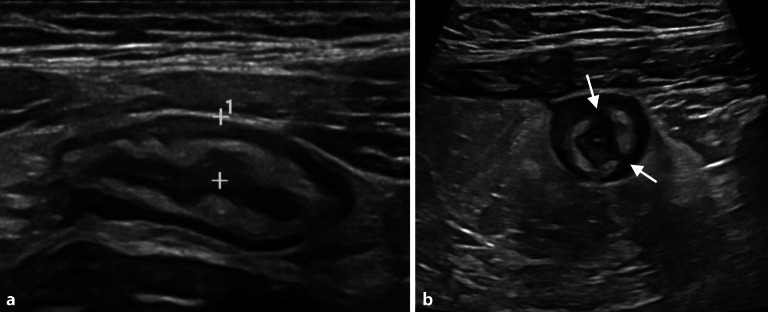
Die Sonographie zeigt eine deutliche Wandverdickung auf > 3 mm (**a**, *Distanzmarken*) mit einer Verdickung der echoreichen Submukosa mit fokalen hypoechogenen Unterbrechungen der Schichtung (**b**, *Pfeile*), hinweisend auf das Vorliegen einer aktiven Entzündung

Des Weiteren wird auf die Wandstratifizierung, die Darstellbarkeit aller Wandschichten und auf umliegende Strukturen (Fettimbibierung, vergrößerte Lymphknoten, freie Flüssigkeit) geachtet (Abb. 3).

Eine internationale Expertengruppe hat kürzlich sonographische Kriterien definiert, die – analog zum Lémann-Score der MRT-Diagnostik – eine Graduierung chronischer Darmwandschäden bei M. Crohn ermöglichen [9].

Verschiedene Scores, wie der Bowel Ultrasound Score (BUSS; [10]) oder der Segmental Activity Score der International Bowel Ultrasound Group (IBUS-SAS; [11]) bei M. Crohn sowie der Milan Ultrasound Score (MUS; [12]) bei Colitis ulcerosa, zeigen eine gute Korrelation mit der endoskopischen und histologischen Krankheitsaktivität. In den meisten Studien erweist sich jedoch die pathologisch verdickte Darmwand (> 3 mm) als ebenso aussagekräftig wie diese teils komplexen Scores, weshalb diese im klinischen Alltag meist nicht verwendet werden.

Trotz zahlreicher Publikationen zum IUS existiert weiterhin keine einheitliche Definition für ein sonographisches Therapieansprechen oder eine Remission. Meistens wird eine sonographische Remission jedoch als Normalisierung der Darmwanddicke (< 3 mm) und Vaskularisierung (Color-Doppler-Signal: 0–1) definiert.

Die **kontrastverstärkte Sonographie**

Kontrastverstärkte Sonographie

ermöglicht eine sowohl qualitative als auch quantitative Analyse der Darmvaskularisation und kann zu einer verbesserten Differenzierung zwischen aktiver Entzündung und fibrotischen Veränderungen beitragen [13]. Rezente Studien befassen sich mit der Anwendung der Shear-wave(Scherwellen)- bzw. der Strain(Kompressions)-**Elastographie**

Elastographie

des Dünndarms zur Diagnostik von Darmwandfibrosen und zur Beurteilung eines Therapieansprechens [14]. Dabei fehlen jedoch prospektive, randomisierte Studien zur abschließenden Bewertung des klinischen Stellenwerts dieser Methode.

#### Merke

Die Auswahl der geeigneten Sonden ist entscheidend für eine vollständige Darmsonographie. Niedrig frequente Sonden ermöglichen die Darstellung tieferliegender Strukturen, während hoch frequente Sonden eine detaillierte Beurteilung der Darmwand erlauben. Eine spezielle Vorbereitung oder Nahrungskarenz vor der Untersuchung ist in der Regel nicht erforderlich. Eine gefüllte Harnblase kann jedoch die Sonographie des Rektums erleichtern.

### Computertomographie

Die CT ist ein zentrales Verfahren zur Diagnostik akuter Dünndarmerkrankungen. Sie ermöglicht eine rasche und umfassende Beurteilung sowohl der Darmwand als auch des umgebenden Mesenteriums und kann dadurch entscheidend zur Ursachenfindung akuter klinischer Symptome wie Schmerzen, Darmobstruktion oder gastrointestinaler Blutung beitragen.

Abseits der Akutdiagnostik können Pathologien des Dünndarms im Rahmen der Abklärung anderer Erkrankungen gefunden werden.

In Notfallsituationen wird häufig ein **Mehrphasenprotokoll**

**Abb. 4:**
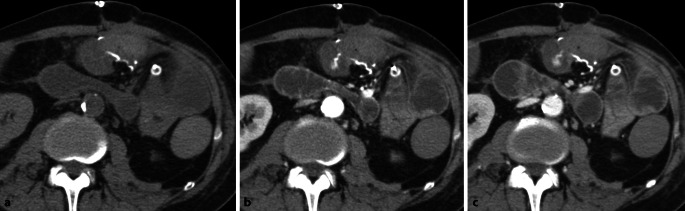
Patient mit postoperativem Hämoglobinabfall und Bauchschmerzen nach Dünndarmteilresektion: Die native Serie (**a**) zeigt Nahtmaterial im Anastomosenbereich. In der arteriellen Phase (**b**) ist ein Kontrastmittelextraluminat direkt neben der Anastomose erkennbar mit geringem Pooling in der venösen Phase (**c**), beweisend für das Vorliegen einer aktiven arteriellen Blutung

Mehrphasenprotokoll

mit Akquisitionen in arterieller und portalvenöser Kontrastmittelphase eingesetzt, um vaskuläre Komplikationen, Blutungsquellen oder ischämische Veränderungen detektieren zu können. Bei Verdacht auf eine akute Blutung des Dünndarms kann eine zusätzliche native Phase zur Differenzierung von hyperdensem Darminhalt von intraluminalen Kontrastmittelfahnen hilfreich sein (Abb. 4). Die arterielle Phase ist dabei am sensitivsten für die Detektion einer aktiven Blutung [15].

Mittels **Dual-energy-CT**

Dual-energy-CT

kann die native Serie durch eine virtuelle Non-contrast-Serie ersetzt werden. Dabei wird mithilfe der spektralen Differenzierung der aufgenommenen Röntgenenergien das Kontrastmittel digital aus den kontrastmittelverstärkten CT-Bildern herausgerechnet. Das ermöglicht eine Reduktion der Strahlendosis um bis zu 30 % [16]. Zusätzliche Postprocessing-Techniken wie virtuelle monochromatische Niedrig-keV-Bilder erhöhen den Jodkontrast. Jod-Maps können die Detektion von Kontrastmittelfahnen erleichtern.

Standardrekonstruktionen beinhalten koronale und sagittale multiplanare Reformationen. Zusätzliche dreidimensionale (3-D) Volume-rendering-Rekonstruktionen ermöglichen die gezielte Darstellung von Aneurysmen oder Gefäßstenosen.

Die **orale**

oral verabreichtes Kontrastmittel

**Abb. 5:**
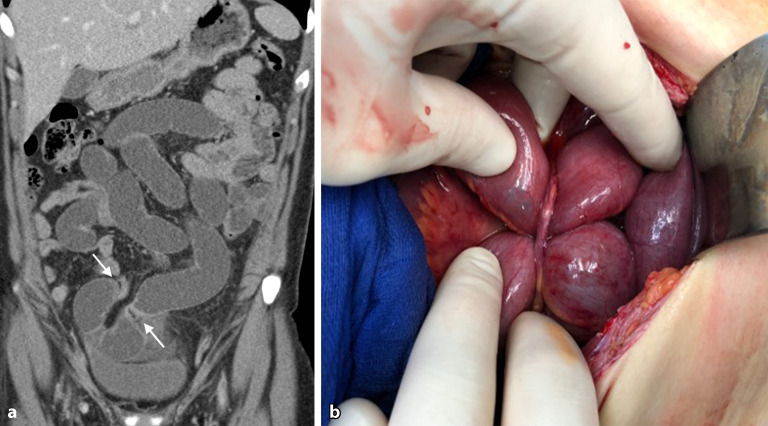
Patient mit akuten Bauchschmerzen bei stattgehabter Appendektomie vor einigen Jahren: Die umgehend durchgeführte Computertomographie des Abdomens (**a**) zeigt distendierte Darmschlingen mit 2 Kalibersprüngen im Unterbauch (*Pfeile*) und nachfolgend kollabiertem Darm, wodurch sich der hochgradige Verdacht auf eine Inkarzeration einer Dünndarmschlinge durch eine Bride mit konsekutiver Dünndarmobstruktion ergab. **b** Intraoperativer Situs, der die Bride bestätigte. (Courtesy: Dr. C. Bichler, Medizin. Univ. Wien)

Verabreichung von **Kontrastmittel**

oral verabreichtes Kontrastmittel

ist derzeit Gegenstand kontroverser Diskussionen. In der Akutsituation ist sie oft nicht praktikabel oder diagnostisch nicht notwendig, wie beispielsweise bei Verdacht auf Darmobstruktion, zumal pathologisch distendierte prästenotische Darmschlingen bereits einen ausreichenden natürlichen Kontrast aufweisen (Abb. 5).

**Abb. 6:**
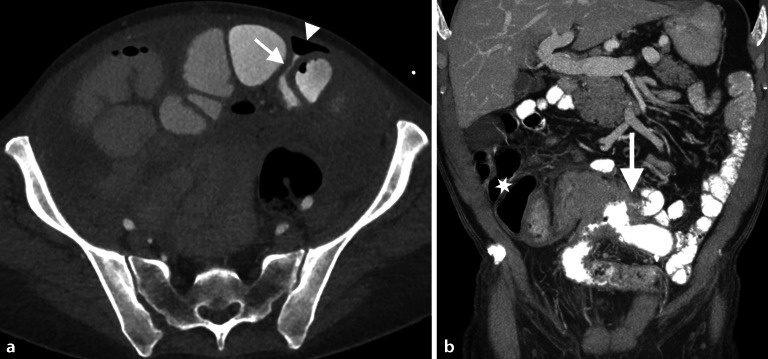
Zwei unterschiedliche Patienten mit positivem oralen Kontrastmittel. **a** Patientin mit peritoneal metastasiertem Appendixkarzinom nach hypertherm intraperitonealer Chemotherapie (*HIPEC*): Aufgrund steigender Entzündungsparameter und abdominaler Schmerzen erfolgte eine Computertomographie (CT), die ein kleines Dünndarmleak (*Pfeil*) mit begleitender extraluminaler Gasansammlung im ventralen Abdomen zeigt. **b** Patient nach Ileosigmoidostomie bei ausgedehntem Morbus Crohn und Bauchschmerzen sowie progredienter Diarrhö: Das koronale CT-Bild zeigt eine ausgedehnte Raumforderung im Bereich der Anastomose (verifiziert als Lymphom) und eine Fistelbildung (*Pfeil*); zu beachten ist das fehlende intraluminale Kontrastmittel vor der Anastomose durch die fistelbedingte Umgehung (*Stern*)

Der großzügigere Einsatz von positivem Kontrastmittel wird allerdings von einigen Experten zunehmend befürwortet, wobei die vergleichsweise geringe Verlängerung der Untersuchungszeit um etwa 30–40 min als klinisch vertretbar angesehen wird [17]. Bei bestimmten Fragestellungen, wie dem postoperativen Nachweis eines Dünndarmlecks, ist die Gabe von positiv oralem Kontrastmittel grundsätzlich indiziert (Abb. 6).

Bei Verdacht auf eine intestinale Fistel oder auf Interloop-Abszesse kann alternativ analog zur CT-Enterographie ein neutrales Kontrastmittel eingesetzt werden, um die Detailanalyse der Darmwandstrukturen nicht zu beeinträchtigen.

Bei Untersuchungen zur gezielten Dünndarmevaluation wie beispielsweise der Enterographie ist die orale Gabe von neutralem Kontrastmittel in einer Menge von 1000–1500 ml essenziell. Intravenöses Kontrastmittel in der enteralen (45 s) oder venösen Parenchymphase dient der besseren Abklärung der Oberbauchorgane [18].

#### Merke

Positives orales Kontrastmittel kann die Beurteilung einer pathologischen Kontrastmittelaufnahme der Darmwand erschweren, Pathologien maskieren und die Detektion von luminalen Blutungen verhindern. Daher sind neutrale Kontrastmittel bei unklaren Dünndarmerkrankungen zu bevorzugen.

### Magnetresonanztomographie

Die MRT des Dünndarms mittels Enterographie hat sich neben der Sonographie als zentrale Methode zur Diagnose entzündlicher Darmerkrankungen etabliert. Tab. 1 zeigt ein typisches Untersuchungsprotokoll ohne und mit intravenösem Kontrastmittel (Tab. 1).

**Tab. 1:** Protokoll einer Magnetresonanzenterographie (1,5 oder 3 T)

*Minimalprotokoll*				
**Sequenz**	**Orientierung**	**Fettsuppression**	**i.v. Kontrastmittel**	**Beurteilbare Pathologien**
SSFP GE	Coronal	−	−	Übersicht
T2 FSE	Axial + coronal	−	−	Anatomie, Wanddicke, Stenosen, Fisteln
T2 FSE	Axial oder coronal	+	−	Mural/perienterales Ödem
*Optionale Sequenzen (zumindest 2 oder 3)*				
3‑D T1 GE	Axial + coronal	+	−/+	KM-Aufnahme der Darmwand, penetrierende Komplikationen
DWI (b-values 50 + 600–800)	Axial	n/a	n/a	Detektion/Verifikation einer Entzündung, Abszess
Cine balanced SSFP GE	Coronal	–	n/a	Motilität, fixierte Strikturen

*SSFP* „steady-state free precession“, *GE* „gradient echo“, *FSE* „fast spin echo“, *3‑D* dreidimensional, *DWI* „diffusion-weighted imaging“, *KM* Kontrastmittel, *n/a* nicht anwendbar

Neben der luminalen Kontrastierung [19] ist v. a. bei Untersuchungsprotokollen mit intravenösem Kontrastmittel die Gabe eines Spasmolytikums nötig, um bei bewegungssensitiven Sequenzen Artefakte zu vermeiden. Die European Society of Gastrointestinal and Abdominal Radiology (ESGAR) empfiehlt daher die intravenöse Applikation von Butylscopolamin [18], wobei die Applikation, geteilt in Form von 2 halben Dosen nach den Motilitätssequenzen und direkt vor der Gadoliniumgabe, die Effektivität steigert und zusätzliche diagnostische Genauigkeit liefern kann [20]. Die Enterographie wird zunehmend auch mit **Kurzprotokollen**

Kurzprotokollen

ohne intravenöse Kontrastmittel durchgeführt, insbesondere für Verlaufskontrollen. Das vereinfacht die Untersuchung und reduziert Kosten, Patientenbelastung und potenzielle Risiken einer Kontrastmittelgabe. Solche Protokolle kombinieren T2-gewichtete und diffusionsgewichtete Bildgebungen („diffusion-weighted imaging“, DWI) zur Detektion von Entzündungsprozessen. Eine T2-gewichtete Sequenz mit Fettunterdrückung ist dabei unerlässlich für die Beurteilung eines muralen oder perienteralen Ödems, das ein sensitives Zeichen einer aktiven Entzündung ist. Funktionelle Komponenten wie bewegungsbasierte („motility“) Sequenzen (z. B. „cine-balanced steady-state free precession“, bSSFP), erlauben die Beurteilung einer eingeschränkten Peristaltik oder einer segmentalen Darmparalyse („frozen bowel“), wie sie in aktiven Entzündungsphasen auftreten können.

Eine Metaanalyse von 39 Studien [21] hat bestätigt, dass Kurzprotokolle bei guter luminaler Distension und hochwertigen DWI- oder Motilitätssequenzen vollständigen Kontrastmittelprotokollen diagnostisch ebenbürtig sind. Einschränkungen bestehen bei der Detektion perforierender bzw. penetrierender Komplikationen sowie bei sehr frühen oder subtilen Veränderungen [21].

### Hybridverfahren

Die Hybridbildgebung mittels PET-CT und PET-MR hat in den letzten Jahren zunehmend an Bedeutung für die Diagnostik von Dünndarmerkrankungen gewonnen. Durch die Kombination funktioneller PET-Information mit der hohen anatomischen Detailauflösung der CT- oder MR-Bildgebung wird eine differenzierte Charakterisierung sowohl entzündlicher als auch neoplastischer Veränderungen ermöglicht. In der onkologischen Bildgebung stellt die ^**68**^**Ga-DOTANOC-PET-CT**

68Ga-DOTANOC-PET-CT

ein zentrales Verfahren zur Lokalisation und Stadieneinteilung neuroendokriner Dünndarmneoplasien dar [22] und unterstützt die Planung einer möglichen Radionuklidtherapie mit ^177^Lu-DOTA-Konjugaten. Bei gastrointestinalen Stromatumoren (GIST) hat sich die **FDG(Fluordesoxyglukose)-PET-CT**

FDG-PET-CT

als wertvolle Ergänzung zur Detektion sekundärblastomatöser Veränderungen etabliert [23]. Bei chronisch-entzündlichen Darmerkrankungen ermöglicht die Hybridbildgebung (insbesondere die PET-MRT) mittels FDG die Erfassung der metabolischen Aktivität entzündlicher Dünndarmsegmente [24] und mittels neuer Tracer (z. B. fibroblastenaktivierender Proteininhibitor [FAPI]) durch spezifische Bindung an aktivierte Myofibroblasten der Darmwand eine Quantifizierung fibrotischer Veränderungen [25]. Die klinische Verfügbarkeit der PET-MRT ist derzeit jedoch auf spezialisierte Zentren beschränkt, mit einem Einsatz überwiegend im Rahmen wissenschaftlicher Fragestellungen.

## Algorithmus für Darmwandpathologien

Die einzelnen Wandschichten des Dünndarms (Mukosa, Submukosa, Muscularis propria, Subserosa und Serosa) können bei gesunden Patient:innen nicht voneinander differenziert werden. Bei guter Distension beträgt die Wanddicke des Dünndarms höchstens 1–2 mm, in kollabiertem Zustand nimmt sie zu und kann bis zu 3 mm betragen. Für die Beurteilung pathologischer Wandveränderungen ist der Vergleich mit unauffälligen Dünndarmabschnitten ähnlichen Distensionsgrades hilfreich.

Es können verschiedene Muster einer Darmwandverbreiterung differenziert werden. Sie ergeben sich aus dem morphologischen Aspekt des Querschnittsbilds und dem muralen Kontrastmittelverhalten. Zusammen mit weiteren Faktoren wie der Länge des betroffenen Segments, der topographischen Lokalisation betroffener Darmabschnitte und dem Vorliegen begleitender periintestinaler Veränderungen lassen sich Schlüsse auf die zugrunde liegende Erkrankung ziehen.

Ein strukturierter Zugang mit systematischer Analyse dieser radiologischen Muster und Zeichen kann die Diagnosefindung wesentlich unterstützen.

### Darmwanddicke

Pathologien des Dünndarms sind meist durch eine Verdickung der Darmwand auf mehr als 3 mm charakterisiert, während eine Ausdünnung typischerweise im Rahmen einer Ischämie auftritt. Die Wandverdickung allein ist allerdings ein unspezifisches radiologisches Zeichen und kann entzündliche, vaskuläre, neoplastische, metabolische oder posttherapeutische Ursachen haben. Mild ausgeprägte Verdickungen (3–4 mm) finden sich häufiger bei entzündlichen Prozessen, wohingegen ausgeprägte Verdickungen von über 9 mm eher für tumoröse Läsionen sprechen.

#### Merke

Kollabierte Dünndarmsegmente oder Spasmen können pathologische Befunde wie Wandverdickungen vortäuschen. In der MRT sollten daher alle Sequenzen sorgfältig beurteilt werden, da sich der Distensionsgrad im zeitlichen Verlauf der Untersuchung verändern kann. Darüber hinaus sollte auch auf perienterale Begleitpathologien geachtet werden, die einen indirekten Hinweis auf das Vorliegen von Erkrankungen liefern können.

### Darmwandmuster

Unterschiedliche Muster der muralen Kontrastmittelaufnahme und ödematöse, fettige oder gashaltige Einlagerungen in einzelne Wandabschnitte können zu charakteristischen Schichtungsphänomenen führen [26]. Diese werden im Querschnittsbild begutachtet und sind differenzialdiagnostisch hilfreich.

Unterschieden werden die weiße und die graue Darmwand, das Wasser-Halo- und das Fett-Halo-Zeichen sowie die schwarze Darmwand.

Eine **weiße Darmwand**

Weiße Darmwand

resultiert aus einer homogenen diffusen muralen Kontrastmittelaufnahme infolge einer Vasodilatation, die einen bildmäßig hellen, „weißen“ Aspekt der Darmwand ergibt. Sie findet sich in akuten Phasen chronisch-entzündlicher Darmerkrankungen, im Rahmen eines Schockgeschehens (Schockdarm), bei Reperfusionsödem nach Ischämie oder auch bei intramuralen Hämatomen aufgrund einer muralen Bluteinlagerung.

Das Bild einer **grauen Darmwand**

Graue Darmwand

ergibt sich hingegen aus einer fehlenden oder reduzierten Kontrastmittelaufnahme. Pathophysiologisch liegen diesem Bild häufig eine Minderperfusion bei Ischämie, chronisch-entzündliche bzw. fibrotische Veränderungen (z. B. M. Crohn) oder eine tumoröse Veränderung (Adenokarzinom, Lymphom) zugrunde.

Das **Wasser-Halo-Zeichen**

Wasser-Halo-Zeichen

oder Schießscheibenzeichen in Form von konzentrischen Ringen findet sich typischerweise bei einem Ödem der Darmwand. Die murale Flüssigkeitseinlagerung führt in Schnittbilduntersuchungen zu einer Zwei- oder Dreischichtung der Darmwand, die durch eine verstärkte Kontrastmittelaufnahme der Mukosa und ggf. der Lamina muscularis propria/serosa hervorgehoben wird. Das submuköse Ödem stellt sich in der CT als vergleichsweise hypodensere mittlere Schicht dar. In der MRT ist es in T2-fettunterdrückten Sequenzen stark hyperintens. Im Querschnitt ergibt sich der Aspekt von konzentrischen Ringen, die an eine Schießscheibe erinnern (Target- oder Schießscheibenzeichen).

Dieses unspezifische Muster findet sich typischerweise bei akuten Erkrankungen wie infektiöser Enteritis, akuten Schüben eines M. Crohn, ischämischer oder radiogener Enteritis sowie einer Graft-versus-host-Erkrankung („graft-versus-host disease“, GvHD).

**Fetteinlagerungen**

Fetteinlagerungen

in der Submukosa können bei langjährigem M. Crohn, nach Chemo- oder Strahlentherapie und im Rahmen einer Zöliakie auftreten. Sie finden sich aber auch bei gesunden Personen und bei adipösen Menschen.

Die **schwarze Darmwand**

Schwarze Darmwand

(Pneumatosis intestinalis) entsteht durch intramurale Gasansammlungen mit zystischem oder linearem Aspekt. Eine intestinale Pneumatose gilt als potenzielles Alarmsignal für eine kritische Darmischämie, kann jedoch auch bei einer Vielzahl benigner Zustandsbilder mit gestörter Mukosabarriere vorkommen, etwa bei COPD („chronic obstructive pulmonary disease“), Kollagenoasen oder idiopathischen Formen, die bevorzugt das Kolon betreffen [27].

**Abb. 7:**
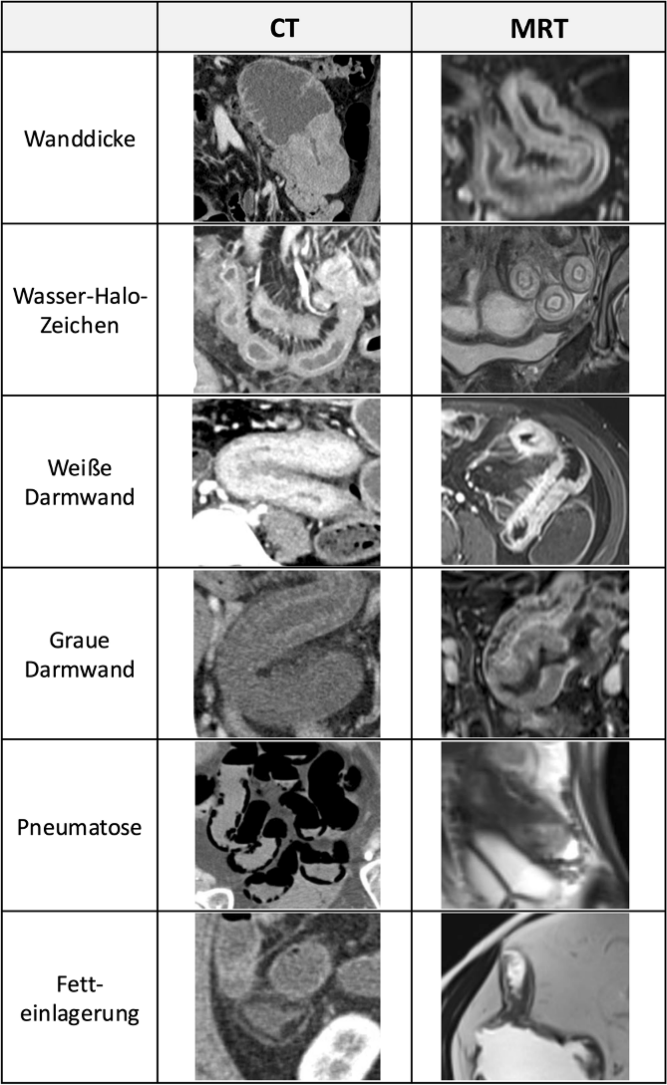
Darmwandmuster in der Computertomographie (*CT*) und in der Magnetresonanztomographie (*MRT*)

Abb. 7 zeigt die verschiedenen Darmwandmuster und ihr Erscheinungsbild in der CT sowie der MRT.

### Länge des betroffenen Segments

Die Längsausdehnung des betroffenen Segments kann wertvolle Hinweise auf die zugrunde liegende Erkrankung liefern.

**Fokale Wandverdickungen**

Fokale Wandverdickungen

mit einer segmentalen Länge von weniger als 5 cm weisen eher auf tumoröse Läsionen hin.

Liegt einer kurzstreckigen Wandverdickung eine fokale entzündliche Veränderung zugrunde, handelt es sich typischerweise um die Entzündung einzelner Divertikel (fokale Divertikulitis) oder seltener um granulomatöse Erkrankungen wie M. Crohn.

**Längere segmentale Veränderungen**

Längere segmentale Veränderungen

von 5–40 cm finden sich häufiger bei Entzündungen wie infektiösen Enteritiden oder ebenfalls bei M. Crohn. Eine Längsausdehnung des betroffenen Darmsegments auf mehr als 40 cm (**diffuse Darmwandveränderung**

Diffuse Darmwandveränderung),

spricht hingegen eher für eine benigne Genese wie Infektionen, Hypalbuminämie, Ischämie, ein Angioödem oder eine GvHD.

### Symmetrie des betroffenen Segments

Eine symmetrische Verdickung der Zirkumferenz der Darmwand weist auf eine Entzündung, ein Ödem, eine Ischämie oder eine Einblutung in die Darmwand hin. Eine exzentrische Wandverdickung hingegen spricht eher für eine maligne Erkrankung. Ausnahmen stellen die mesenterialseitig betonte Entzündungsreaktion bei M. Crohn dar sowie bestimmte Lymphome, die ebenfalls symmetrische Wandveränderungen hervorrufen können.

### Topografische Verteilung

Neben dem Ausmaß der Darmwandverbreiterung, dem spezifischen Muster sowie der Länge des betroffenen Segments ist auch die topographische Verteilung der Veränderungen im Gastrointestinaltrakt für die differenzialdiagnostische Einordnung von entscheidender Bedeutung. Von Relevanz ist dabei, ob multiple Abschnitte des Gastrointestinaltrakts oder lediglich ein einzelnes Segment betroffen sind. Ebenso ist zu berücksichtigen, ob neben dem Dünndarm auch der Dickdarm mitbefallen ist. Bestimmte anatomische Lokalisationen wie beispielsweise die Beteiligung des terminalen Ileums bei M. Crohn können zusätzliche Hinweise auf die zugrunde liegende Pathologie liefern.

### Weite des Dünndarmlumens

In der CT- und MRT-Enterographie mit oraler Distension wird beim Dünndarm allgemein eine Lumenweite von unter 3 cm als physiologisch angesehen. Eine pathologische Dilatation tritt sowohl als Folge einer Obstruktion als auch bei einer Paralyse auf. Patient:innen mit M. Crohn entwickeln im Laufe ihres Lebens in bis zu 50 % eine Striktur mit Symptomen einer Obstruktion [28]. Bisherige Richtlinien definieren eine **prästenotische Dilatation**

Prästenotische Dilatation

als Folge einer signifikanten Stenose ab einer Lumenweite von über 3 cm [29]. Fehlt bei einer signifikanten Wandverdickung mit einer luminalen Einengung (> 50 % im Vergleich zum angrenzenden normalen Darm) die prästenotische Dilatation von mehr als 3 cm, wird von einer wahrscheinlichen Striktur gesprochen [29, 30]. Dieser Grenzwert von 3 cm Lumenweite wird allerdings zunehmend als nicht ausreichend erachtet, und neueste Empfehlungen der Society of Abdominal Radiology (SAR) definieren für CT- und MRT-Untersuchungen eine Lumenweite von über 2,5 cm als diagnostisches Kriterium für eine signifikante prästenotische Dilatation [16].

Im intestinalen Ultraschall wird aufgrund der fehlenden Distension durch orales Kontrastmittel eine Weite von weniger als 2,5 cm als regulär erachtet. Dabei können eine Pendelperistaltik, flüssigkeitsgefüllte Darmschlingen und ein Klaviertastenphänomen zusätzlich typisch für eine Obstruktion sein.

## Auflösung Kasuistik

**Abb. 8:**
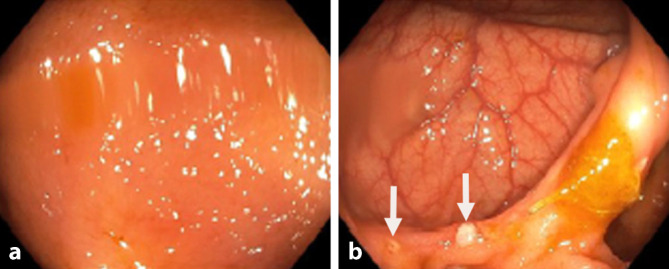
Endoskopische Sicht auf die Anastomosenregion mit **a** unauffälligem neoterminalem Ileum und **b** einzelnen aphtösen Läsionen an der ileokolischen Anastomose (*Pfeile*, **b**)

Die hoch auflösende Darmsonographie des Abdomens zeigte eine lineare echoreiche Struktur, die sich über das blinde Ende der enterokolischen Anastomosenregion erstreckte. Das umgebende Fettgewebe zeigte echoreiche ödematöse Veränderungen. In der weiterführenden CT des Abdomens wurde eine von der Anastomose ausgehende Fistelformation bestätigt. Die Patientin musste erneut operiert werden (erweiterte Ileoaszendostomie). Nach 5 Monaten präsentiert sie sich mit normalisiertem Calprotectin und einem endoskopischen Ansprechen (Rutgeerts-Score i2a; Abb. 8). Mit der leicht verfügbaren Ultraschalluntersuchung konnte der Verdacht einer Fistulierung gestellt werden, mit einer CT-Akutuntersuchung ließ sich dieser Verdacht bestätigen und eine zeitnahe weiterführende chirurgische Sanierung ermöglichen.

## Zusammenfassung

Die radiologische Diagnostik des Dünndarms hat durch technische Innovationen und verbesserte Bildgebungstechniken erheblich an Bedeutung gewonnen. Entscheidend für die diagnostische Qualität sind eine indikationsbasierte Wahl des geeigneten Verfahrens, die standardisierte Durchführung und eine strukturierte Befundung. Radiolog:innen nehmen dabei eine Schlüsselrolle ein, indem sie nicht nur Bilder interpretieren, sondern auch die klinische Fragestellung in den Mittelpunkt stellen und im interdisziplinären Austausch einen essenziellen Beitrag zur Patientenversorgung leisten.

## Fazit für die Praxis

- Eine gezielte und korrekt durchgeführte Schnittbilddiagnostik ermöglicht nicht nur die Diagnose und Charakterisierung von Dünndarmerkrankungen, sondern auch die Beurteilung eines Therapieansprechens im Rahmen von Verlaufsuntersuchungen.
- Der intestinale Ultraschall und die MR(Magnetresonanz)-Enterographie sind die Methoden der Wahl für chronisch-entzündliche Darmerkrankungen, wohingegen die Computertomographie des Abdomens in der Akutdiagnostik den Vorrang hat.
- Die Pathologien des Dünndarms sind eine heterogene Gruppe, deren radiologische Befundkriterien sich häufig überlappen können.
- Eine strukturierte Analyse charakteristischer muraler Veränderungen unter Berücksichtigung der klinischen Anamnese ermöglicht eine gezielte Eingrenzung möglicher Differenzialdiagnosen.

## CME-Fragebogen

Welches Limberg-Stadium beschreibt eine Darmwandverdickung mit vermehrt kurzstreckigen Gefäßen?

Limberg 0

Limberg 1

Limberg 2

Limberg 3

Limberg 4

Welche anatomische Struktur ist in der intestinalen Sonographie der pathologischen Darmwand oft echoreich verdickt?

Muscularis mucosae

Submukosa

Muscularis propria

Lumen

Gefäße

Welches orale Kontrastmittel kommt in der Enterographie zum Einsatz?

Positives Kontrastmittel

Kein Kontrastmittel

Neutrales Kontrastmittel

Negatives Kontrastmittel

Jodhaltiges Kontrastmittel

Welches ist *keine* Indikation zur Anfertigung einer Abdomen-Leeraufnahme?

Verdacht auf intestinale Obstruktion

Lokalisation einer verschluckten Münze

Beurteilung der Kontrastmittelpassage bei V. a. inkomplette Obstruktion

Verlaufskontrolle einer Pneumatosis intestinalis

Ingestion einer Knopfbatterie

Eine 38-jährige Patientin nach allogener Stammzelltransplantation wird mit akuten Bauchschmerzen und Diarrhö in die Notaufnahme gebracht. Eine Abdomencomputertomographie mit intravenösem Kontrastmittel wird durchgeführt. Dort zeigt sich ein Wasser-Halo-Zeichen der Dünndarmwand. Welche der folgenden Diagnosen ist am wahrscheinlichsten?

Akute bakterielle Enteritis

Ischämische Enteritis

Akute Graft-versus-host-Erkrankung

Akuter Schub eines Morbus Crohn

Radiogene Enteritis

Die sog. schwarze Darmwand (Pneumatosis intestinalis) gilt in erster Linie wofür als Alarmsignal?

Kollagenosen

Zöliakie

Kritische Darmischämie

Chronische Strahlenenteritis

Morbus Crohn im Langzeitverlauf

Die Dual-Energy-Computertomographie in der Dünndarmdiagnostik hat folgenden Vorteil:

Die virtuelle Non-contrast-Serie wird zusätzlich zur nativen Serie durchgeführt.

Sie ersetzt die intravenöse Kontrastmittelapplikation.

Virtuelle monochromatische Hoch-keV-Bilder erhöhen den Jodkontrast.

Jod-Maps können bei der Detektion von luminalen Blutungen hilfreich sein.

Metallartefakte können durch niedrig energetische virtuell monochromatische Bilder reduziert werden.

Welche Aussage zur MR(Magnetresonanz)-Enterographie trifft am besten zu?

Buscopan ist erforderlich, um intestinale Bewegungsartefakte zu minimieren.

Intravenöses Kontrastmittel muss zur Diagnostik einer Darmwandverdickung verabreicht werden.

T1-gewichtete Sequenzen stellen die Hauptsequenz zur Evaluation von Dünndarmerkrankungen dar.

Diffusionsgewichtete Sequenzen werden primär zur Detektion eines muralen Ödems eingesetzt.

Coronale T2-gewichtete Spin-echo-Sequenzen werden zunehmend als Motility-Sequenzen in der MR-Enterographie eingesetzt.

Ein 61-jähriger Patient kommt mit Bauchschmerzen und Erbrechen in die Notfallaufnahme. Die Computertomographie (CT) mit intravenösem Kontrastmittel zeigt einen Kalibersprung im linken Oberbauch mit einer kurzstreckigen, asymmetrischen, ausgeprägten Wandverdickung. Welche Diagnose ist am wahrscheinlichsten?

Infektiöse Enteritis

Hypalbuminämie

Angioödem

Maligner Tumor des Dünndarms

Graft-versus-host-Erkrankung

Welche Aussage zur Weite des Darmlumens ist korrekt?

Die Pendelperistaltik in der intestinalen Sonographie ist ein spezifisches Zeichen für eine paralytische Darmobstruktion ohne mechanisches Hindernis.

Eine wahrscheinliche Striktur ist durch eine fehlende Darmwandverdickung definiert.

Bei der intestinalen Sonographie wird aufgrund fehlender Distension mit oralem Kontrastmittel eine Lumenweite < 2,5 cm als normal erachtet.

Die prästenotische Dilatation wird als Lumenweite > 2 cm vor einer Stenose definiert.

Eine Dünndarmstriktur ist eine seltene Komplikation bei Morbus Crohn.
